# Cigarette smoke differentially affects IL‐13‐induced gene expression in human airway epithelial cells

**DOI:** 10.14814/phy2.13347

**Published:** 2017-07-12

**Authors:** Tinne C. J. Mertens, Anne M. van der Does, Loes E. Kistemaker, Dennis K. Ninaber, Christian Taube, Pieter S. Hiemstra

**Affiliations:** ^1^ Department of Pulmonology Leiden University Medical Center Leiden The Netherlands; ^2^ Department of Molecular Pharmacology University of Groningen Groningen The Netherlands

**Keywords:** Asthma, interleukin 13, primary human epithelial cells, T helper 2, whole cigarette smoke

## Abstract

Allergic airways inflammation in asthma is characterized by an airway epithelial gene signature composed of *POSTN*,*CLCA1*, and *SERPINB2*. This Th2 gene signature is proposed as a tool to classify patients with asthma into Th2‐high and Th2‐low phenotypes. However, many asthmatics smoke and the effects of cigarette smoke exposure on the epithelial Th2 gene signature are largely unknown. Therefore, we investigated the combined effect of IL‐13 and whole cigarette smoke (CS) on the Th2 gene signature and the mucin‐related genes *MUC5AC* and *SPDEF* in air–liquid interface differentiated human bronchial (ALI‐PBEC) and tracheal epithelial cells (ALI‐PTEC). Cultures were exposed to IL‐13 for 14 days followed by 5 days of IL‐13 with CS exposure. Alternatively, cultures were exposed once daily to CS for 14 days, followed by 5 days CS with IL‐13. *POSTN*,*SERPINB2*, and *CLCA1* expression were measured 24 h after the last exposure to CS and IL‐13. In both models *POSTN*,*SERPINB2*, and *CLCA1* expression were increased by IL‐13. CS markedly affected the IL‐13‐induced Th2 gene signature as indicated by a reduced *POSTN*,*CLCA1*, and *MUC5AC* expression in both models. In contrast, IL‐13‐induced *SERPINB2* expression remained unaffected by CS, whereas *SPDEF* expression was additively increased. Importantly, cessation of CS exposure failed to restore IL‐13‐induced *POSTN* and *CLCA1* expression. We show for the first time that CS differentially affects the IL‐13‐induced gene signature for Th2‐high asthma. These findings provide novel insights into the interaction between Th2 inflammation and cigarette smoke that is important for asthma pathogenesis and biomarker‐guided therapy in asthma.

## Introduction

Asthma is a syndrome characterized by airway hyperresponsiveness, chronic inflammation, and mucus hypersecretion. Historically asthma, and particularly allergic asthma, has been considered to be mainly driven by a T helper 2 (Th2)‐mediated immune response. However, it is now well recognized that asthma is a heterogeneous disease with different pathophysiological pathways underlying airway inflammation (Wenzel 2012). Molecular phenotyping of diseased airway tissue has the potential to unravel the multiple phenotypes of asthma. Furthermore, it allows the identification of biomarkers associated with specific disease patterns to select patients for personalized targeted therapies.

Approximately 50% of asthmatic patients have Th2‐mediated disease (Woodruff et al. 2009; Wenzel 2012). A Th2‐high subtype of asthma has been described and is associated with increased bronchial epithelial expression of periostin (*POSTN*), serpin B2 (*SERPINB2*), and chloride channel regulator 1 (*CLCA1*), and predicts a beneficial therapeutic response to corticosteroids (Woodruff et al. 2007, 2009). Recently various clinical trials have shown the potential of inhibitors of Th2 inflammation, including monoclonal antibodies against interleukin (IL)‐13, to modulate clinical outcomes in asthma (van Buul and Taube 2015). IL‐13 is produced by Th2 cells and has been shown to have marked effects on airway epithelial cells (Whittaker et al. 2002; Woodruff et al. 2007). IL‐13 is an important mediator for the induction of goblet cell metaplasia in Th2‐mediated asthma and is a central regulator in the epithelial expression of *POSTN*,* SERPINB2*, and *CLCA1* (Woodruff et al. 2007). Periostin, the protein encoded by the *POSTN* gene, is of particular interest as a biomarker, as it is detectable in the circulation and may be useful as a blood biomarker for IL‐13‐activated bronchial epithelial cells. Indeed, there is evidence suggesting that circulating periostin levels may help in the identification of asthma patients that benefit from anti‐IL‐13 treatment (Corren et al. 2011; Noonan et al. 2013; Brightling et al. 2015).

Asthma has a genetic predisposition, but is it recognized that environmental factors are very important in the pathogenesis. An important environmental factor influencing asthma pathogenesis is cigarette smoking. Approximately 20–35% of the world population smokes, with surprisingly similar smoking rates reported in asthmatic patients (Cerveri et al. 2012; To et al. 2012; Thomson et al. 2013). Cigarette smoking has been shown to worsen asthma symptoms, reduce responsiveness to corticosteroid treatment, accelerate lung function decline, and increase exacerbation rates (Polosa and Thomson 2013). Additionally, smoking is strongly predictive for the development of new onset asthma in atopic adults (Polosa et al. 2008). As a history of current or former smoking is present in approximately 20–30% of the asthmatic population (Cerveri et al. 2012; Thomson et al. 2013), cigarette smoking could be considered as one of the most important environmental factors influencing asthma pathogenesis.

We have previously shown that the IL‐13‐induced epithelial Th2 gene signature can be differentially affected by azithromycin treatment, suggesting that IL‐13 induces its gene expression pattern through various pathways (Mertens et al. 2016). Furthermore, a suppressive effect of cigarette smoke on *POSTN* and *SERPINB2* gene expression has previously been suggested based on a study focusing on the presence of a Th2 gene signature in patients with chronic obstructive pulmonary disease (COPD) (Christenson et al. 2015). Surprisingly little is known about the effect of cigarette smoking on IL‐13‐activated airway epithelial cells and the IL‐13‐induced gene expression pattern described for Th2‐high asthma. Therefore, we have investigated, for the first time, the combined effect of whole cigarette smoke exposure and IL‐13 on primary human airway epithelial cells cultured at the air–liquid interface, thus providing novel insights into the interaction between Th2 inflammation and cigarette smoke that is relevant for asthma pathogenesis and biomarker‐guided therapy in asthma.

## Material and Methods

### Cell culture

Human primary bronchial epithelial cells (PBEC) were isolated from macroscopically normal bronchial tissues obtained from lung cancer patients undergoing lobectomy at the Leiden University Medical Center (Leiden, The Netherlands). Primary tracheal epithelial cells were isolated from residual tracheal and main stem bronchial tissue from lung transplant donors postmortem at the University Medical Center Groningen (Groningen, the Netherlands). Use of lung tissue that became available for research within the framework of patient care was in line with the “Human Tissue and Medical Research: Code of conduct for responsible use” (2011) (www.federa.org) that describes the no‐objection system for coded anonymous further use of such tissue. Therefore, individual written or verbal consent is not applicable. Details on isolation of PBEC (Mertens et al. 2016) and PTEC (Kistemaker et al. 2015) were described previously. During 14 days of differentiation, cell culture medium was replaced every 2 days.

Cultured PBEC and PTEC were used for generation of mucociliary differentiated cultures by differentiation at the air–liquid interface (ALI) as described previously (Mertens et al. 2016). Briefly, PBEC and PTEC at passage 2 were cultured submerged on semipermeable transwell inserts with 0.4 *μ*m pore size (Corning Costar, Cambridge, MA) that were coated with a mixture of bovine serum albumin, collagen type 1, and fibronectin. Once full confluence was reached, apical medium was removed and PBEC or PTEC were used for subsequent experimental exposures.

### Experimental design

**Figure 1:**
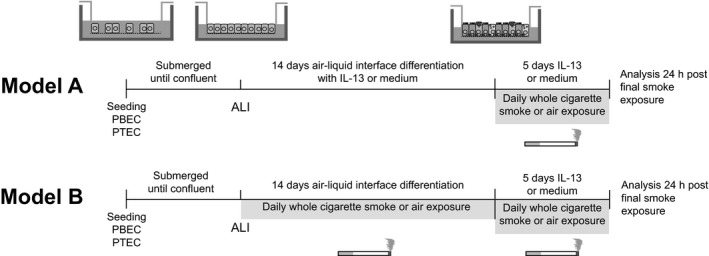
Exposure setup to investigate the effect of whole cigarette smoke exposure on the IL‐13‐induced expression pattern in human bronchial and tracheal epithelial cells. ALI, air–liquid interface; IL‐13, interleukin‐13; PBEC, primary bronchial epithelial cells; PTEC, primary tracheal epithelial cells.

Two experimental models were used to investigate the effects of whole cigarette smoke exposure on the IL‐13‐induced expression pattern (Fig. 1). In exposure Model A, ALI‐PBEC or ALI‐PTEC were grown to confluence, and cultured for 14 days at the ALI in the presence of 1 or 2.5 ng/mL IL‐13 which was added in the basolateral compartment of the transwell insert, followed by an additional 5 days once daily whole cigarette smoke or air exposure in the presence or absence of continued treatment with 1 or 2.5 ng/mL recombinant human IL‐13 (Peprotech, Rocky Hill, CT) which was added in the basolateral compartment of the transwell insert. In Model B, ALI‐PBEC or ALI‐PTEC were grown to confluence, and next cultured at the ALI and exposed once daily to whole cigarette smoke or air for 14 days, followed by an additional 5 days once daily whole cigarette smoke or air exposure in the presence or absence of continued treatment with 10 ng/ml IL‐13 that was added to the basolateral compartment of the transwell insert. For both models, ALI‐PBEC or ALI‐PTEC were rinsed apically with 200 *μ*L PBS, 4 h prior to whole cigarette smoke or air exposure. During 14 days of exposure, cell culture medium was replaced every two days. During the last 5 days of exposure, medium was refreshed daily, directly after whole cigarette smoke or air exposure. Twenty‐four hours after the last whole cigarette smoke or air exposure, cells were lysed for RNA or protein extraction, and basal medium was collected and stored at −20°C until further use.

### Whole cigarette smoke exposure

ALI‐PBEC or ALI‐PTEC were exposed to cigarette smoke generated from 3R4F reference cigarettes (University of Kentucky, Lexington, KY) in a whole cigarette smoke exposure model adapted from Beisswenger et al. (2004) as described previously (Amatngalim et al. 2015). In brief, ALI cultures were placed into modified hypoxic chambers (Billups Rothenberg, Del Mar, CA), localized inside an incubator at 37°C and 5% CO_2_. Whole cigarette smoke derived from one cigarette, or air as negative control, was permeated inside the respective exposure chamber using a continuous flow of 1 l/min for a period of 4–5 min. After exposure, residual smoke inside the exposure chamber was removed by flushing the chambers with air derived from the incubator for a period of 10 min. After smoke or air exposure, cell culture medium was refreshed and cells were incubated at 37°C and 5% CO_2_.

### RNA isolation, RT, and qPCR

Total RNA was extracted using the Maxwell 16 LEV simplyRNA Tissue Kit (Promega, Leiden, The Netherlands) and quantified using the Nanodrop ND‐1000 UV‐visible spectrophotometer (Nanodrop Technologies, Wilmington, DE). For cDNA synthesis, 1 *μ*g of total RNA was reverse transcribed using oligo(dT) primers and Moloney murine leukemia virus (M‐MLV) polymerase (Promega) at 37°C. Primer sequences are listed in Table 1. RPL13A and ATP5B were used as reference genes following selection by the Genorm method (Vandesompele et al. 2002). All quantitative PCRs (qPCRs) were carried out in triplicate on a CFX‐384 real‐time PCR detection system (Bio‐Rad Laboratories, Veenendaal, The Netherlands) with the use of SYBR green (Bio‐Rad). Bio‐Rad CFX manager 3.1 software (Bio‐Rad) was used to calculate arbitrary gene expression by using the standard curve method.

**Table 1:** Primer sequences with gene names and NCBI gene ID used in present study

Gene	Primer sequence	NCBI gene ID
*POSTN*	F: GAC CGT GTG CTT ACA CAA ATT G	10631
	R: AAG TGA CCG TCT CTT CCA AGG	
*SERPINB2*	F: TCC TGG GTC AAG ACT CAA ACC	5055
	R: CAT CCT GGT ATC CCC ATC TAC AG	
*CLCA1*	F: ATG GCT ATG AAG GCA TTG TCG	1179
	R: TGG CAC ATT GGG GTC GAT TG	
*MUC5AC*	F: CCT TCG ACG GAC AGA GCT AC	4586
	R: TCT CGG TGA CAA CAC GAA AG	
*SPDEF*	F: ATG AAA GAG CGG ACT TCA CCT	25803
	R: CTG GTC GAG GCA CAG TAG TG	
*RPL13A*	F: AAG GTG GTG GTC GTA CGC TGT G	23521
	R: CGG GAA GGG TTG GTG TTC ATC C	
*ATP5B*	F: TCA CCC AGG CTG GTT CAG A	506
	R: AGT GGC CAG GGT AGG CTG AT	

### Periostin and mucin 5AC ELISA

Periostin protein expression was measured in medium collected from the basolateral compartment of the transwell 24 h after the last whole cigarette smoke or air exposure. Periostin ELISA was performed according to the manufacturer's instruction (R&D Systems Europe Ltd., Abingdon, United Kingdom). For Mucin 5AC protein expression, cells were lysed in RIPA buffer 24 h after the last whole cigarette smoke or air exposure according to the manufacturer's instruction (Thermo Fisher Scientific, Breda, The Netherlands). Lysate was diluted in bicarbonate coating buffer without azide and incubated in a NUNC maxisorp ELISA plate (Thermo Fisher Scientific) at 37°C until dry. Plates were washed and nonspecific binding sites were blocked with PBS/2% (w/v) BSA (Sigma‐Aldrich Chemie BV, Zwijndrecht, The Netherlands) for 2 h at room temperature, followed by 2 h incubation with mouse anti‐MUC5AC (1:200; 45M1; Thermo Fisher Scientific) in PBS/0.05% Tween‐20 (v/v) (Sigma‐Aldrich) at room temperature. Next, plates were washed with PBS/0.05% Tween‐20 and incubated for 1 h with goat anti‐mouse HRP (1:2000, Dako Denmark A/S, Glostrup, Denmark) at room temperature. Plates were developed using tetramethylbenzidine‐hydrogen peroxidase solution and the reaction was stopped with 2.5 mol/L H_2_SO_4_. Absorbance was measured at 450 nm using a Microplate reader (iMark; Bio‐Rad Laboratories, Hercules, CA) and Microplate Manager Software (version 6.3, Bio‐Rad).

### SDS‐PAGE and western blot

Protein RIPA lysates were diluted (1:1 [v/v]) in sodium dodecyl sulfate (SDS) sample buffer containing 4% (w/v) SDS (Sigma‐Aldrich), 20% (v/v) glycerol (Merck), 0.8% (w/v) dl‐dithiothreitol (Sigma‐Aldrich), 0.5 mol/L Tris pH 6.8 and 0.003% (w/v) bromophenol blue (Sigma‐Aldrich), heated for 5 min at 100°C, and applied on a 4–15% SDS‐PAGE gel (Mini‐PROTEAN TGX, Bio‐Rad). Next, proteins were blotted on a Trans‐Blot Turbo Mini PDVF membrane using the Trans‐Blot Turbo Transfer System (Bio‐Rad). Nonspecific binding sites were blocked in Tris‐buffered saline (TBS)/0.05% (v/v) Tween‐20 containing 5% (w/v) skimmed milk. Membranes were probed with rabbit‐anti‐CLCA1 (1:1000; EPR12254‐88; Abcam, Cambridge, United Kingdom), rabbit‐anti‐SERPINB2 (1:1000; ab47742; Abcam), rabbit‐anti‐POSTN (1:1000, ab14041, Abcam), or GAPDH (1:1000; 14C10; Cell Signaling Technologies, Leiden, The Netherlands) in 5% (w/v) BSA TBS/0.05% (v/v) Tween‐20 overnight at 4°C. Next, membranes were incubated with anti‐rabbit IgG HRP‐linked antibody (1:10,000, Cell Signaling Technologies) in blocking buffer for 1 h and binding was revealed using enhanced chemiluminescence substrate (Thermo Fisher Scientific).

### Statistical analysis

Graphs were made and statistical analysis was performed in GraphPad PRISM 6.0 (GraphPad Software Inc., La Jolla, CA). Differences were explored by one‐way ANOVA with Dunnett's test. Data are shown as means ± SEM of cultures derived from several donors and differences were considered significant at *P* < 0.05.

## Results

### An established IL‐13‐induced gene expression pattern is differentially affected by whole cigarette smoke exposure

**Figure 2:**
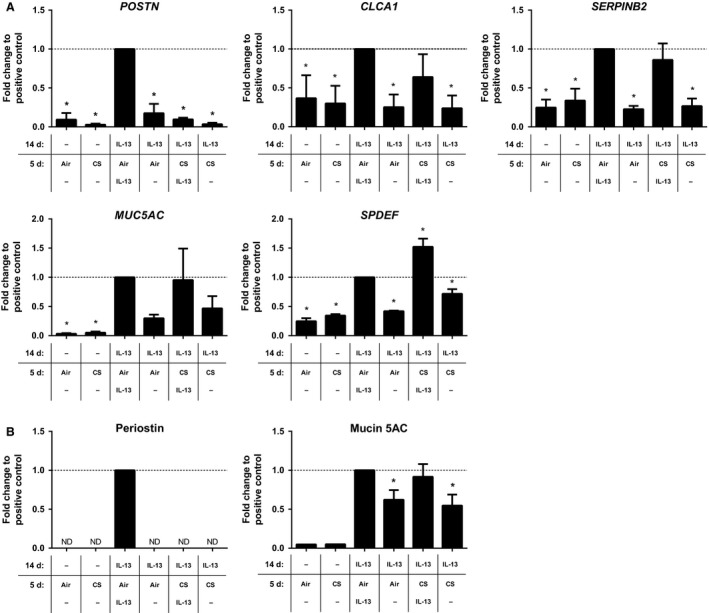
Effect of whole cigarette smoke exposure on IL‐13‐induced gene expression in ALI‐PBEC. ALI‐PBEC were differentiated with IL‐13 (1 ng/mL) for 14 days followed by an additional 5 days with IL‐13 (1 ng/mL) in the presence of air or CS exposure. *MUC5AC*,*POSTN*,*SERPINB2*,*SPDEF*, and *CLCA1* gene expression (A) were assessed by qRT‐PCR; periostin and mucin 5AC proteins (B) were assessed by ELISA. Results are expressed as mean ± SEM fold change compared to IL‐13‐exposed ALI‐PBEC exposed to air (indicated by a horizontal dashed line) with *n* = 4 independent donors. *P < 0.05. ALI, air–liquid interface; CS, whole cigarette smoke exposure; IL‐13, interleukin‐13; PBEC, primary bronchial epithelial cells.

First, we investigated the effect of whole cigarette smoke (CS) exposure on an established IL‐13‐induced Th2 gene expression pattern in ALI‐PBEC. To this end, ALI‐PBEC were differentiated for 14 days in the presence of IL‐13 to establish this Th2 gene signature, followed by 5 days exposure to CS in the presence or absence of IL‐13 (Fig. 1, Model A). IL‐13‐differentiated ALI‐PBEC showed indeed significantly increased expression of the Th2 signature genes *POSTN*,* SERPINB2*, and *CLCA1*, in addition to the goblet cell metaplasia‐related genes *MUC5AC* and *SAM*, pointed domain containing ETS transcription factor (*SPDEF*) (Fig. 2A). IL‐13‐differentiated ALI‐PBEC exposed to CS in the presence of IL‐13 had significantly reduced *POSTN* expression compared to the IL‐13‐differentiated ALI‐PBEC exposed to air in the presence of IL‐13. In contrast, *MUC5AC* and *SERPINB2* expression remained unaffected, whereas *SPDEF* expression was additively increased in CS‐exposed IL‐13‐differentiated ALI‐PBEC compared to air exposure (Fig. 2A). Cessation of IL‐13 exposure during the last 5 days reduced IL‐13‐induced gene expression back to baseline expression levels, suggesting that the effect of IL‐13 does not persist (Fig. 2A). Cigarette smoke exposure has previously been shown alter methylation patterns in airway epithelial cells (Beane et al. 2007). As mucin 5AC and periostin are both important biomarker proteins, we validated our gene expression findings for *MUC5AC* and *POSTN* using ELISA (Fig. 2B). Mucin 5AC protein expression appeared to be lowered upon IL‐13 exposure cessation; however, 5 days seems insufficient time to return protein levels to baseline expression levels. Additionally, periostin protein could only be detected in ALI‐PBEC that were continuously exposed to IL‐13 without the presence of CS (Fig. 2B).

**Figure 3:**
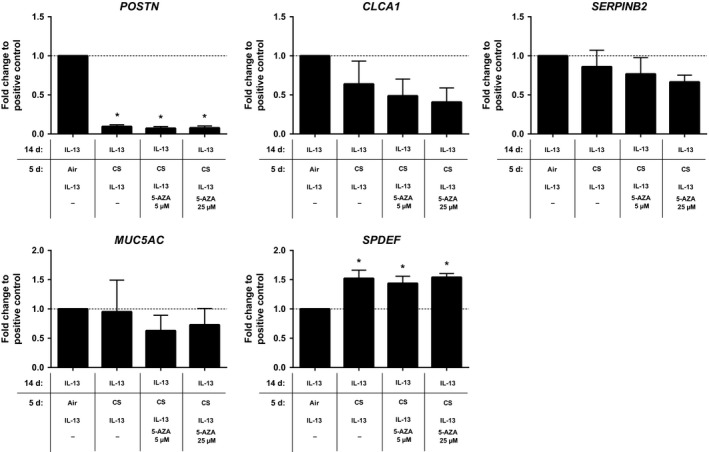
Whole cigarette smoke exposure does not affect the IL‐13‐induced Th2 gene signature through promoter methylation. ALI‐PBEC were differentiated with IL‐13 (1 ng/mL) for 14 days followed by an additional 5 days with IL‐13 (1 ng/mL) in the presence of air or whole cigarette smoke exposure with or without 5‐azacytidine. *MUC5AC*,*POSTN*,*SERPINB2*,*SPDEF*, and *CLCA1* gene expression (A) were assessed by qRT‐PCR; POSTN and MUC5AC proteins (B) were assessed by ELISA. Results are expressed as mean ± SEM fold change compared to IL‐13‐exposed ALI‐PBEC exposed to air (indicated by a horizontal dashed line) with *n* = 4 independent donors. *P < 0.05. ALI, air–liquid interface; 5‐AZA, 5‐azacytidine; CS, whole cigarette smoke exposure; IL‐13, interleukin‐13; ND, not detected; PBEC, primary bronchial epithelial cells.

To investigate whether the effect of CS on IL‐13‐induced *POSTN* expression resulted from CS‐induced DNA methylation effects, we daily treated CS‐exposed IL‐13‐differentiated ALI‐PBEC with the demethylating compound 5‐azacytidine (5 or 25 *μ*mol/L) during the last 5 days of CS exposure. However, 5‐azacytidine treatment during CS exposure was unable to restore *POSTN* expression levels (Fig. 3).

### Whole cigarette smoke exposure differentially affects IL‐13‐induced responsiveness

**Figure 4:**
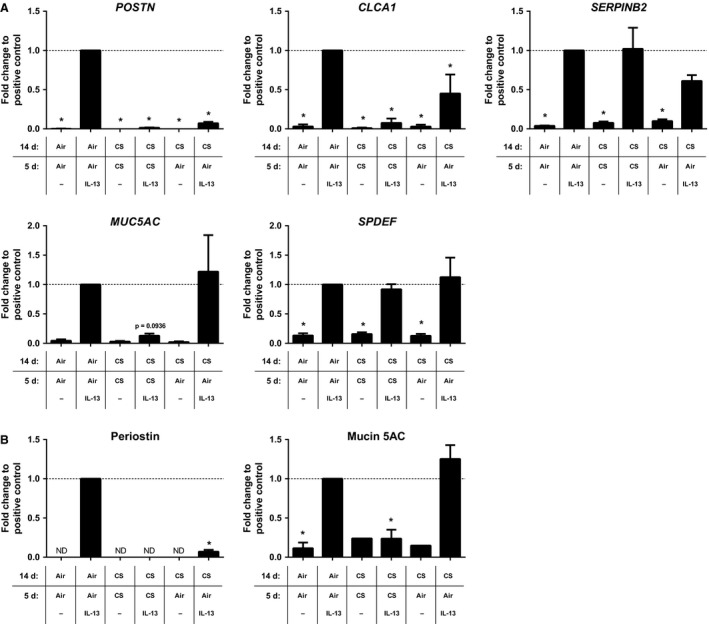
Effect of IL‐13 on ALI‐PBEC differentiated in the presence of air or whole cigarette smoke exposure. ALI‐PBEC were exposed to once daily air or CS exposure during differentiation for 14 days, followed by an additional 5 days with IL‐13 (10 ng/mL) in the presence of air or CS exposure. *MUC5AC*,*POSTN*,*SERPINB2*,*SPDEF*, and *CLCA1* gene expression (A) were assessed by qRT‐PCR; periostin and mucin 5AC proteins (B) were assessed by ELISA. Results are expressed as mean ± SEM fold change compared to IL‐13‐exposed ALI‐PBEC exposed to air (indicated by a horizontal dashed line) with *n* = 4 independent donors. *P < 0.05. ALI, air–liquid interface; CS, whole cigarette smoke exposure; IL‐13, interleukin‐13; PBEC, primary bronchial epithelial cells.

Our results showed a noticeably differential effect of CS exposure on an established IL‐13‐induced gene expression pattern in ALI‐PBEC. Next, we investigated whether chronic CS exposure in ALI‐PBEC affected the ability of IL‐13 to promote the expression of these genes. To this end, we differentiated ALI‐PBEC with daily CS exposure or air as a control for 14 days, followed by another 5 days of daily CS (or air) exposure in the absence or presence of IL‐13 (10 ng/mL, added in the basal chamber) as depicted in Figure 1, Model B. Pilot results indicated that short‐term IL‐13 exposure of ALI‐PBEC differentiated in the presence of CS exposure induced STAT6 phosphorylation, an important downstream mediator of IL‐13‐induced changes, to the same extent as control differentiated ALI‐PBEC, suggesting that CS‐differentiated ALI‐PBEC are still able to respond to IL‐13 (results not shown). Control ALI‐PBEC exposed for 5 days to IL‐13 displayed an increased expression of the Th2‐signature genes *POSTN*,* SERPINB2*, and *CLCA1*, in addition to an increase in the goblet cell metaplasia‐related genes *MUC5AC* and *SPDEF* (Fig. 4A). In contrast, CS‐differentiated ALI‐PBEC exposed to IL‐13 had significantly reduced *POSTN* and *CLCA1* expression and a trend for reduced *MUC5AC* expression (*P* = 0.094), whereas *SERPINB2* and *SPDEF* expression remained unaffected compared to IL‐13‐exposed control ALI‐PBEC (Fig. 4A). These data indicate that upon CS exposure, IL‐13 is unable to promote the Th2‐signature gene expression to a similar extend as it does in air‐exposed controls.

We next investigated whether CS‐induced reduction of *POSTN*,* CLCA1*, and *MUC5AC* expression would return to IL‐13‐stimulated control levels upon cessation of CS exposure. After 14 days of daily CS exposure, CS‐differentiated ALI‐PBEC were exposed for 5 additional days to air in the presence or absence of IL‐13. Results showed that upon CS cessation, both *POSTN* and *CLCA1* gene expression remained diminished in the presence of IL‐13; however, *MUC5AC* expression was fully restored to the level of IL‐13‐incubated air‐exposed control ALI‐PBEC (Fig. 4A). The findings for mucin 5AC and periostin were confirmed on protein level using ELISA (Fig. 4B). Overall, these results further support that CS exposure significantly affects IL‐13‐induced gene expression patterns in ALI‐PBEC.

### Regional differences in the lung do not affect responses to cigarette smoke and IL‐13

**Figure 5:**
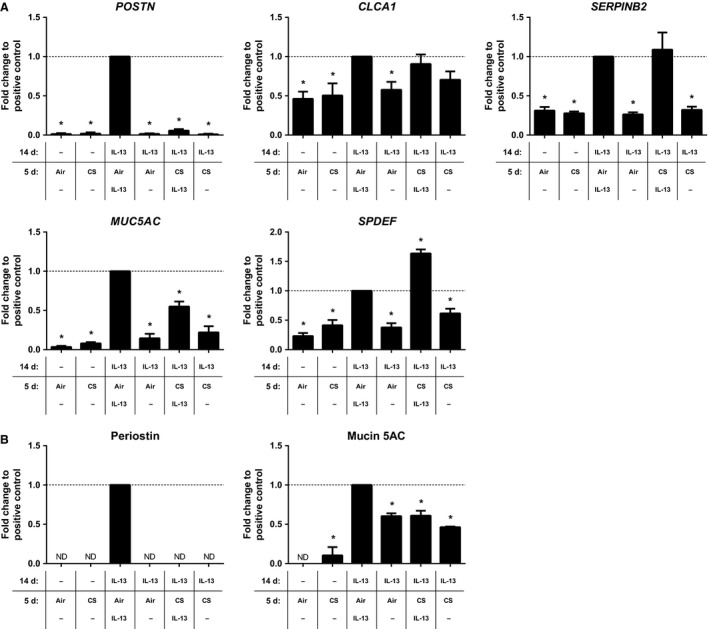
Effect of whole cigarette smoke exposure on IL‐13‐induced gene expression in ALI‐PTEC. ALI‐PTEC were differentiated with IL‐13 (1 ng/mL) for 14 days followed by an additional 5 days with IL‐13 (1 ng/mL) in the presence of air or CS. *MUC5AC*,*POSTN*,*SERPINB2*,*SPDEF*, and *CLCA1* gene expression (A) were assessed by qRT‐PCR; periostin and mucin 5AC proteins (B) were assessed by ELISA. Results are expressed as mean ± SEM fold change compared to IL‐13‐exposed ALI‐PTEC exposed to air (indicated by a horizontal dashed line) with *n* = 4 independent donors. *P < 0.05. ALI, air–liquid interface; CS, whole cigarette smoke exposure; IL‐13, interleukin‐13; PTEC, primary tracheal epithelial cells.

**Figure 6:**
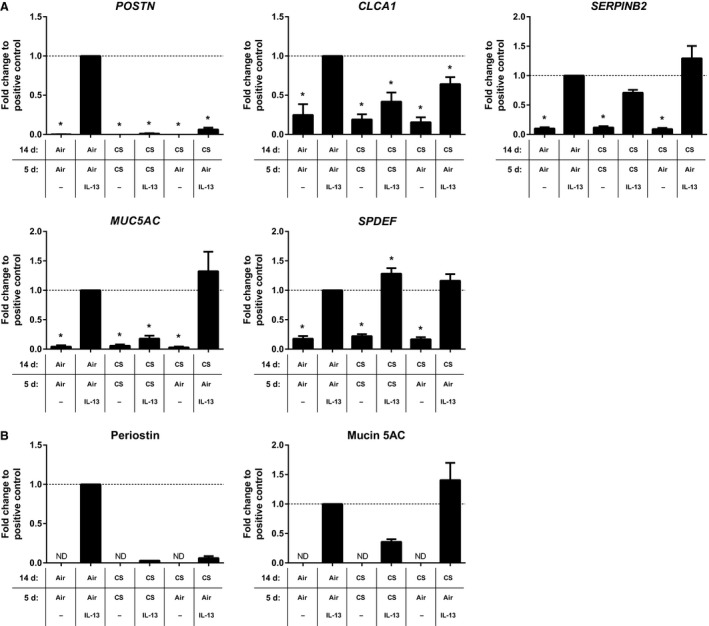
Effect of IL‐13 on ALI‐PTEC differentiated in the presence of whole cigarette smoke exposure. ALI‐PTEC were exposed to daily air or CS exposure during differentiation for 14 days, followed by an additional 5 days with IL‐13 (10 ng/mL) in the presence of air or CS. *MUC5AC*,*POSTN*,*SERPINB2*,*SPDEF*, and *CLCA1* gene expression (A) were assessed by qRT‐PCR; periostin and mucin 5AC proteins (B) were assessed by ELISA. Results are expressed as mean ± SEM fold change compared to IL‐13‐exposed ALI‐PTEC exposed to air (indicated by a horizontal dashed line) with *n* = 4 independent donors. *P < 0.05. ALI, air–liquid interface; CS, whole cigarette smoke exposure; IL, interleukin; ND, not detected; PTEC, primary tracheal epithelial cells.

Primary tracheal epithelial cells (PTEC) are more easily accessible for biomarker studies due to their anatomic location compared to bronchial epithelial cells. Furthermore, tracheal epithelial cells have previously been shown to have a similar biological response to CS exposure compared to the small airway epithelium (Turetz et al. 2009). To investigate whether ALI‐PTEC show similar responses to ALI‐PBEC following combined IL‐13 and CS exposure, we exposed ALI‐PTEC according to Models A and B (Fig. 1). ALI‐PTEC responded similar compared to ALI‐PBEC, with a few exceptions (Figs. 5, 6). In addition to reduced *POSTN* expression by CS‐exposed IL‐13‐differentiated ALI‐PBEC, *MUC5AC* was also significantly reduced in ALI‐PTEC compared to air‐exposed controls (Figs. 5A, 6A), suggesting a slightly stronger disturbance of the Th2 gene signature by CS in ALI‐PTEC compared to the ALI‐PBEC. Gene expression data for mucin 5AC and periostin were confirmed at the protein level in ALI‐PTEC using ELISA (Fig. 5B, 6B). Taken together, our results suggest that CS differentially affects the IL‐13‐induced expression in ALI‐PTEC. Moreover, ALI‐PTEC can be used as an alternative model for ALI‐PBEC to study the effects of IL‐13 and CS exposure with regard to the IL‐13‐induced Th2 gene signature.

## Discussion

The present results show that chronic whole cigarette smoke (CS) exposure differentially affects the IL‐13‐induced gene expression pattern in primary bronchial and tracheal epithelial cells cultured at the air‐liquid interface. Although IL‐13‐induced *POSTN* expression was lowered upon subsequent CS exposure, *MUC5AC*,* CLCA1*, and *SERPINB2* expression remained unaffected and *SPDEF* expression was further increased. Conversely, IL‐13‐responsiveness of primary airway epithelial cells was also severely affected by CS. Differentiation of airway epithelial cells in the presence of CS followed by IL‐13 exposure resulted in reduced expression of *POSTN*,* CLCA1*, and *MUC5AC*, whereas *SERPINB2* and *SPDEF* expression remained unaffected. Cessation of CS exposure in the presence of IL‐13 was insufficient to restore *POSTN* and *CLCA1* expression, while *MUC5AC* expression was fully restored. Together, these data suggest that CS affects, even upon cessation, the Th2 gene signature that has been suggested to distinguish Th2‐high and Th2‐low patients.

The clinical effects of smoking in asthmatic patients have been well described. However, little is known about the effect of cigarette smoking on the molecular phenotype that has been suggested for Th2‐high asthma. Several mouse models of allergic airway inflammation have reported a suppressive effect of cigarette smoke on Th2‐mediated inflammation, including goblet cell metaplasia (Melgert et al. 2004; Robbins et al. 2005; Trimble et al. 2009; Hizume et al. 2012; Tilp et al. 2016). A suppressive effect of cigarette smoke on *POSTN* and *SERPINB2* expression could be expected based on observations on the presence of a Th2 gene signature in a subset of smoking and nonsmoking COPD patients (Christenson et al. 2015). Among the genes that comprise the Th2 gene signature, periostin is of particular interest as it can be detected in serum, thus serving as an easy accessible biomarker to distinguish Th2‐high from Th2‐low asthma patients. Furthermore, high serum periostin levels have been shown to predict therapy response to anti‐IL‐13 treatment (Corren et al. 2011; Hanania et al. 2013; Brightling et al. 2015). In our exposure models, we noticed a marked decrease in periostin mRNA and protein levels upon CS exposure. These data are also in line with a recently published study showing that serum periostin levels are lower in smoking asthmatics compared to nonsmoking controls (Thomson et al. 2015). Indeed, the current study shows the effect of smoking at a cellular level, in part, explaining the results observed in the aforementioned patient study. In addition, the present study also shows that CS exposure cessation failed to restore IL‐13‐induced *POSTN* expression levels, indicating persistence of the effect of CS exposure on IL‐13 responsiveness. Several studies have indicated a persistent effect on gene expression profiles in former smokers even several years after smoke cessation, suggesting the involvement of smoking‐induced epigenetic mechanisms (Beane et al. 2007; Chari et al. 2007; Zhang et al. 2008). A long‐lasting effect after smoking cessation in asthmatics on corticosteroid responsiveness has also been suggested by the observation of an attenuated response to corticosteroid treatment in former smokers with asthma (Chaudhuri et al. 2006). To investigate whether DNA methylation was involved in the observed effects of CS exposure on periostin expression, we used the demethylating agent 5‐aza during CS exposure. However, treatment with 5‐aza failed to prevent the CS‐induced modulation of *POSTN* and *CLCA1* expression, suggesting that DNA methylation is not pivotal in the persistence of decreased expression after CS exposure. However, we cannot formally exclude that possibility that another demethylating agent would have prevented this CS‐induced modulation of gene expression.

Both CS and IL‐13 have been linked to goblet cell metaplasia in airway epithelial cells in vitro and in vivo. In Th2‐mediated asthma, goblet cell metaplasia and associated MUC5AC overexpression are mainly attributed to the presence of IL‐13. IL‐13 induces SPDEF and CLCA1 expression, both essential genes involved in the development of IL‐13‐induced MUC5AC expression (Zhen et al. 2007; Yu et al. 2010; Alevy et al. 2012). Goblet cell metaplasia is increased in smokers, and several studies using cigarette smoke have shown the induction of MUC5AC (Wright et al. 1983; Chari et al. 2007; Cortijo et al. 2011; Iwashita et al. 2012; Wu et al. 2012; Schamberger et al. 2015). Most studies have focused on the effects of cigarette smoke extract rather than whole cigarette smoke to induce goblet cell metaplasia. We observed an increase in *SPDEF* expression, a gene previously shown to be important in the development of goblet cell metaplasia (Park et al. 2007; Chen et al. 2009). In contrast, the present data showed no increase in *MUC5AC* expression in airway epithelial cells exposed to CS. Together, these data suggest that smoking induces a first “hit” for the development of goblet cell metaplasia in smokers, but that an extra stimulus from, for example, underlying tissue inflammation may be required for the development of mucus hypersecretion following goblet cell metaplasia. In our experimental setup, exposure to CS reduced IL‐13‐induced epithelial markers of goblet cell metaplasia. This reduced *MUC5AC* and *CLCA1* expression in CS‐differentiated airway epithelial cells exposed to IL‐13 may be explained by the presence of heme oxygenase 1. Cigarette smoke has previously been shown to induce heme oxygenase 1 expression in our whole cigarette exposure model setup (Zarcone et al. 2016). Heme oxygenase 1 has been shown to inhibit IL‐13‐induced‐MUC5AC expression and more recently, this process was shown to be associated with reduced CLCA1 expression in human bronchial epithelial cells (Almolki et al. 2008; Mishina et al. 2015).

The relevance of our findings is further enhanced by the use of primary epithelial cells derived from multiple donors and from multiple anatomical locations instead of the use of cell lines. In addition, we used cells that were differentiated at the air–liquid interface to allow mucociliary differentiation, instead of cell lines that do not differentiate and display other abnormalities. Finally, we used freshly prepared mainstream whole cigarette smoke containing both the gaseous and particulate components instead of the widely used aqueous extracts of CS. Although quantification of the exposure to whole smoke is difficult, in our view it does provide a more accurate representation of cigarette smoke exposure (gaseous and particulate constituents) compared to aqueous extracts. Bronchial epithelial cells were derived from macroscopically normal resected tissue obtained during surgery for lung cancer from patients that were largely (ex)smokers. Furthermore, epithelial cells from asthmatics were not available to further evaluate the effect of CS on a clinically established Th2 signature. We considered the possibility that cigarette smoke also likely modulates epithelial gene expression induced by cytokines other than IL‐13 that are known to be increased in asthma, such as IL‐17. However, we focused on IL‐13 because it has been well described that the gene expression pattern observed in airway epithelial cells from patients with allergic asthma is well reflected by IL‐13 treatment of cultured primary airway epithelial cells (Woodruff et al. 2007, 2009).

In conclusion, our results indicate that CS differentially affects the IL‐13‐induced expression profile including the recently described epithelial 3 gene signature for Th2‐high asthma. The observation that CS markedly reduces IL‐13‐induced *CLCA1* and *POSTN* expression, which does not recover after CS cessation, is an important finding for biomarker‐guided therapy in asthma since especially periostin is considered as an emerging biomarker for Th2 inflammation. Possibly, periostin may not be a good biomarker for Th2 inflammation in asthmatics that smoke. The observation that CS is able to reduce *MUC5AC* expression but not *SPDEF* expression remains to be further elucidated. Collectively, our results provide novel insight in the interaction between Th2 inflammation and cigarette smoke that is relevant for asthma pathogenesis and biomarker‐guided therapy in asthma.

## Conflict of Interest

The authors declare no conflict of interest.
